# New weapons explosive exhibits persistent toxicity in plants

**DOI:** 10.1038/s41477-024-01863-0

**Published:** 2024-11-28

**Authors:** Nicola C. Oates, Edward R. Nay, Timothy J. Cary, Elizabeth L. Rylott, Neil C. Bruce

**Affiliations:** 1 Centre for Novel Agricultural Products, Department of Biology, University of York, York, UK; 2 Engineer Research and Development Center, Cold Regions Research and Engineering Laboratory, Biogeochemical Sciences Branch, US Army Corps of Engineers, Hanover, NH USA

**Keywords:** Plant physiology, Abiotic

## Abstract

Explosives are widespread, toxic and persistent environmental pollutants. 2,4-Dinitroanisole (DNAN) is being phased in to replace 2,4,6-trinitrotoluene (TNT) in munitions. Here we demonstrate that only low levels of DNAN are detoxified in *Arabidopsis*, leaving it to remain as a substrate for monodehydroascorbate reductase 6 mediated chronic phytotoxicity. Enhancing the potential for environmental toxicity, DNAN is readily transported to the aerial tissues exposing this toxin to herbivores and the wider food chain.

## Abstract (web summary)

This study reports the mechanism of phytotoxicity by the explosive 2,4-dinitroanisole (DNAN) that is being phased in to replace TNT in munitions. Only low levels of DNAN are detoxified by plants, causing chronic toxicity and potential exposure to herbivores.

## Main

The insensitive munition 2,4-dinitroanisole (DNAN) is being increasingly used as an alternative to 2,4,6-trinitrotoluene (TNT) in explosive preparations as it can be cast and melted more safely. A major component of military munitions worldwide, TNT is classified by the US Environmental Protection Agency as a Group C (possible human) carcinogen and toxic to all organisms tested^1^. Furthermore, TNT is not substantially mineralized in the environment, and still present at munition factories and disposal sites dating back to World Wars I and II^1^. The costs of remediating munition constituents from military training ranges in the United States alone is between US$16 billion and US$165 billion^2^, and it is imperative that replacement munitions do not make this situation worse. High (>100 mg kg^−1^) concentrations of TNT in soil are devastating to surrounding vegetation and microorganisms^1^, but inhibitory effects on plants and invertebrates have been observed at as low as 3–4 mg kg^−1^ TNT^1^. The toxicity and detoxification of TNT by plants is well established^1^, and this knowledge has been utilized to engineer robust plant systems to phytoremediate explosives pollution^1^. Given the predicted scale of use as a replacement for TNT, there is an urgent need to understand the toxicity of DNAN in plants and whether plants are capable of detoxifying concentrations that are likely to occur at contaminated sites.

Monodehydroascorbate reductase 6 (MDHAR6) has been determined as the primary cause of TNT phytotoxicity in *Arabidopsis thaliana* (Arabidopsis)^3^. Dual-targeted to mitochondria and plastids, MDHAR6 reduces TNT to a nitro-radical, with the concurrent oxidation of NADH. The radical spontaneously autoxidizes back to TNT, producing a superoxide radical. As TNT is not depleted, this continues in a futile cycle that exhausts NADH and produces superoxides. These superoxides and downstream hydrogen peroxide and other radicals react with, and thus damage, cellular components such as mitochondria, DNA and cell membranes^4^. Given the similarities in structure, we hypothesized that DNAN would elicit similar detoxification strategies to TNT in plants and reasoned that MDHAR6 would mediate toxicity in a manner similar to TNT.

**Fig. 1:**
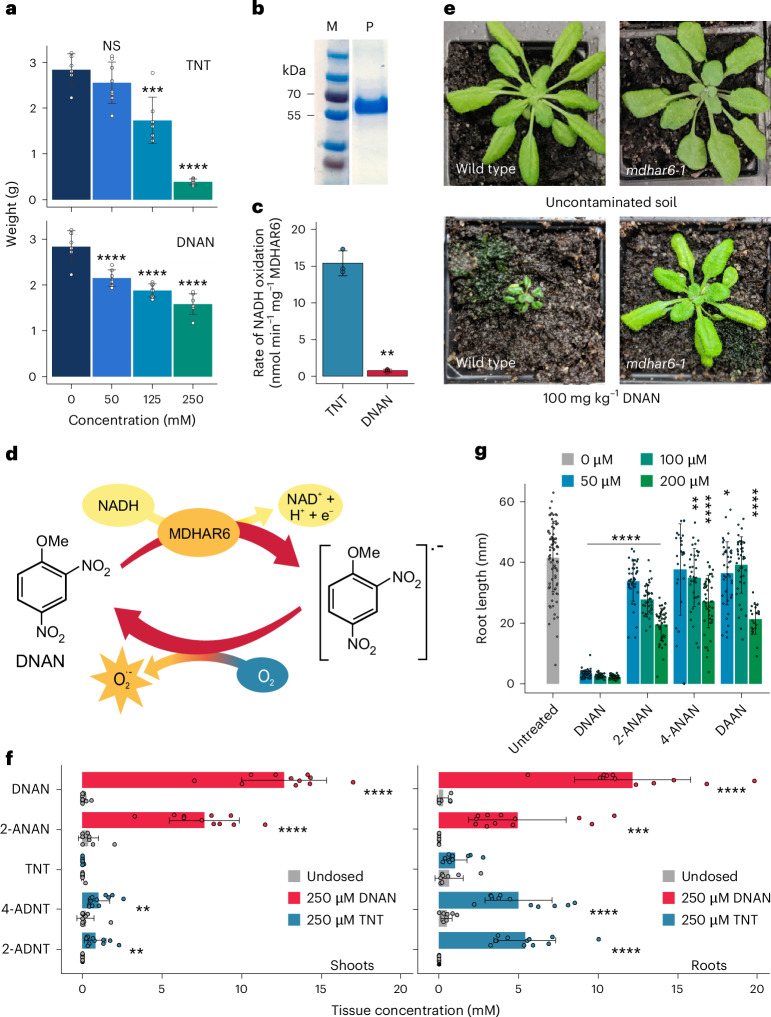
Phytotoxicity and root:shoot partitioning of TNT, and DNAN and intermediates. **a**, Fresh weight of 2-week-old Arabidopsis plants grown in shake flasks, 7 days post dosing (*n* = 8, mean ± s.d.). Statistical significance testing, compared to untreated, was performed using ANOVA followed by Tukey’s post hoc test. **b**, Sodium dodecyl sulfate–polyacrylamide gel electrophoresis (SDS–PAGE) gel showing the recombinant expression and purification of MDHAR6 protein (P), with molecular weight markers (M). **c**, Activity of purified MDHAR6 using TNT and DNAN substrates (*n* = 3, mean ± s.d.); statistical comparison was performed using two-sided *t*-test. **d**, Schematic showing the suggested mode of DNAN phytotoxicity. **e**, Appearance of 5-week-old wild-type and *mdhar6-1* Arabidopsis plants grown on uncontaminated soil and soil contaminated with 100 mg kg^−1^ DNAN. **f**, Levels of DNAN, TNT and intermediates in tissue extracts from 4-week-old, hydroponically grown Arabidopsis 4 days after dosing with 250 µM explosive (*n* ≥ 8, mean ± s.d.). **g**, Root lengths of 14-day-old seedlings germinated and grown on agar plates containing DNAN, 2-ANAN, 4-ANAN or DAAN (*n* ≥ 24, mean ± s.d.). Statistical comparison was performed using one-way ANOVA followed by Tukey’s post hoc test. *P < 0.05, **P < 0.01, ***P < 0.001, ****P < 0.0001. All data and *P* values can be found in the Source data file.

**Extended Data Fig. 1:**
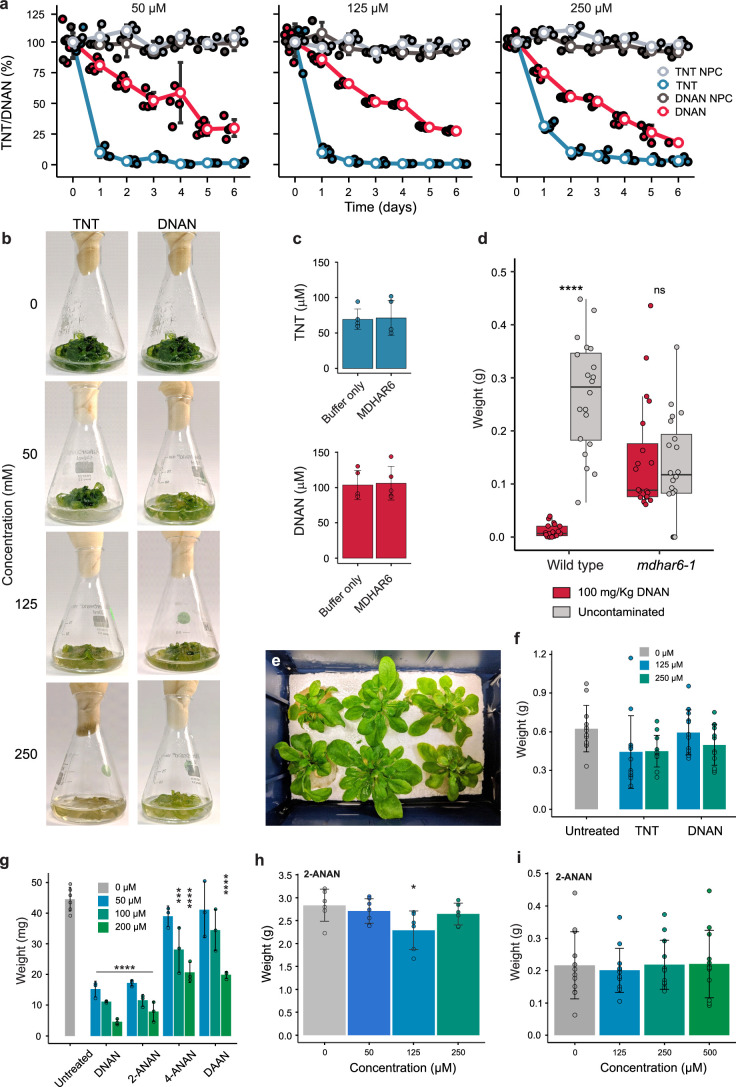
Phytotoxicity of TNT, and DNAN and intermediates. (**a**) Depletion of xenobiotic from shake flask media (n = 5, mean ± SD). (**b**) Appearance of two-week-old Arabidopsis plants grown in shake flasks, seven days post-dosing (**c**) Levels of TNT and DNAN remaining after a 24-hour incubation using purified MDHAR6 in the presence of an NADH recycling system (n = 5, mean ± SD). Concentrations of DNAN and TNT were compared by t-test between MDHAR6 and buffer only reactions. (**d**) Aerial tissue biomass of five-week-old wild type (WT) and *mdhar6-1* Arabidopsis plants grown on uncontaminated soil, and soil contaminated with 100 mg kg^−1^ DNAN (n = 20 plants, mean ± SD). Statistical significance was performed by two-sided t-test. (**e**) Appearance of the hydroponic system and (**f**) levels of DNAN, TNT and intermediates in tissue extracts from four-week-old, hydroponically grown Arabidopsis four days after dosing with 250 µM explosive (n ≥ 8, mean ± SD). (**g**) biomass of seedlings (n ≥ 3, mean ± SD). (**h**) Fresh weight biomass of two-week-old Arabidopsis plants grown in shake flasks and dosed for seven days with 2-ANAN (n ≥ 6, mean ± SD). (**i**) Fresh weight biomass of four-week-old Arabidopsis plants, dosed for seven days with 2-ANAN (n = 12, mean ± SD). Statistical analysis was performed by one-way ANOVA and Tukey’s post hoc test, * p < 0.05, ** p < 0.01, *** p < 0.001, **** p < 0.0001. All data and p values can be found in the Source data file.

To evaluate the relative phytotoxicity of DNAN and TNT, we dosed 2-week-old liquid-culture-grown Arabidopsis plants with a range of DNAN or TNT concentrations that relate to those found on contaminated sites^5–7^ and concentrations of TNT used in previous studies^3,8,9^ for comparison. At 7 days post dosing, at the highest (250 µM) concentration, TNT had the greatest phytotoxic effect (Fig. 1a and Extended Data Fig. 1a,b). Interestingly, at the lowest concentrations tested (50 µM), only DNAN significantly reduced (*P* = 0.003) plant fresh weight compared with the undosed control. Samples taken from the surrounding media revealed that while most of the TNT was depleted within the first 24 h, DNAN remained for considerably longer (Extended Data Fig. 1c). This prolonged exposure probably results in the chronic toxicity observed in lower concentrations of DNAN.

We tested the activity of purified MDHAR6 towards DNAN (Fig. 1b), which was confirmed through the oxidation of NADH, although at a markedly slower rate than that observed with TNT substrate (*P* = 0.0041; Fig. 1c). To determine whether DNAN is depleted, the concentration was measured after a 24-h incubation with MDHAR6 (Extended Data Fig. 1c). Neither the concentration of TNT nor that of DNAN was affected (*P* = 0.89 and 0.85, respectively), indicating that the reaction establishes a futile cycle as depicted in Fig. 1d. To assess the contribution of MDHAR6 to DNAN toxicity in planta, Arabidopsis *mdhar6-1* lines were grown in soil contaminated with 100 mg kg^−1^ DNAN. *mdhar6-1* plants grew notably better than wild type on DNAN-contaminated soil (Fig. 1e), producing aerial biomasses that were not significantly different from those when grown on uncontaminated soil (*P* = 0.62; Extended Data Fig. 1d). This result confirms that MDHAR6 has a physiological role in the phytotoxicity of DNAN in Arabidopsis plants.

To further understand the localization of DNAN in plants and whether transformation intermediates are produced, we established a hydroponic system as shown in Extended Data Fig. 1e. Four-week-old Arabidopsis plants were dosed with 250 µM DNAN or TNT and the tissue analysed at 4 days post exposure. In agreement with previous studies^8,9^, TNT was quickly transformed to both 2-ADNT and 4-ADNT intermediates and localized almost exclusively in the root tissues (Fig. 1f). In contrast, DNAN localized to both root and aerial tissue and was only partially reduced to 2-amino-4-nitroanisole (2-ANAN). After 7 days, plants dosed with DNAN or TNT were weighed; there was no corresponding change in tissue weight for the DNAN-dosed plants compared to untreated controls (Extended Data Fig. 1f). This result is probably because these plants were grown for longer than in the previous shake flask experiments in Fig. 1a and thus larger when first dosed with TNT or DNAN, facilitating enhanced nitroreduction.

The transformation of DNAN to 2-ANAN would be beneficial to the plant if 2-ANAN is less phytotoxic than DNAN. To compare the toxicity of 2-ANAN against DNAN, seeds were germinated and seedlings grown in agar plates containing DNAN and ANAN. After 14 days, severe root length inhibition was observed in the DNAN-treated seedlings relative to those dosed with the transformation intermediates (Fig. 1g; at 50 µM, there was an 81% reduction in root length for DNAN, compared with 7% for 2-ANAN, *P* = 4.9 × 10^−10^ and 2.5 × 10^−5^, respectively). However, when the seedlings were weighed, the biomasses of DNAN and 2-ANAN-treated plants were equally reduced (38% and 35%, *P* = 1.0 × 10^−8^ and 4.8 × 10^−8^, respectively; Extended Data Fig. 1g). To further understand the toxicity of 2-ANAN, 2-week-old plants were dosed in shake flasks (as shown in Extended Data Fig. 1b,h) and 4-week-old plants were grown hydroponically (Extended Data Fig. 1e,i), then dosed with 2-ANAN for 7 days. Apart from a small (*P* = 0.38) difference in biomass in shake flasks with 125 μM 2-ANAN, there was no effect on plant biomass, indicating that 2-ANAN toxicity is developmental stage dependent. Seedling toxicity of the chemically related intermediates 4-ANAN and 2,4-diaminoanisole (DAAN) was found to be less than that of 2-ANAN (Fig. 1g).

**Fig. 2:**
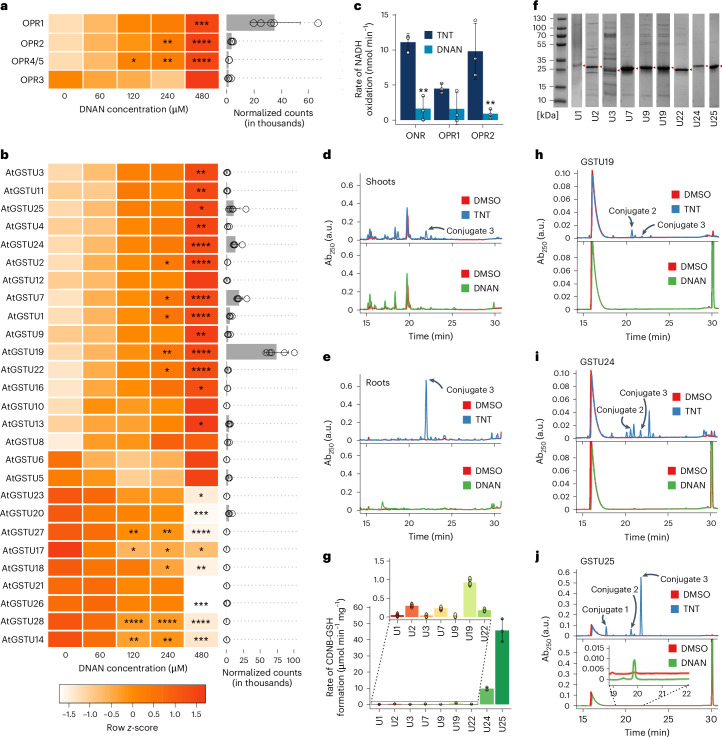
Role of OPRs and GSTs in DNAN transformation. **a**,**b**, Upregulation of OPRs (**a**) and Tau-class GSTs (**b**) from a transcriptomics study of 2-week-old, liquid-culture-grown plants dosed with DNAN for 6 h (*n* = 5, mean ± s.d.). Heat map (left) showing the relative expression levels across different concentrations of DNAN, in terms of *z*-score. The bar plot (right) depicts the normalized counts for each gene at 480 μM DNAN. **c**, Activity of purified recombinant OPRs towards TNT and DNAN (*n* = 3, mean ± s.d.). Unless otherwise stated, statistical significance testing was performed using one-way ANOVA followed by Tukey’s post hoc test. **d**,**e**, Analysis for the presence of GST conjugates in plant tissue of 2-week-old liquid-culture-grown plants, dosed with 250 μM TNT or DNAN for 7 days. HPLC traces are shown for shoots (**d**) and roots (**e**). Conjugate peaks are labelled on each trace, as previously identified^12,13^. HPLC analysis was repeated on 12 separate plants for each condition. **f**, SDS–PAGE gels of recombinantly expressed and purified GSTs (marked with red arrows). **g**, Conjugating activity of recombinant GSTs with CDNB substrate (*n* = 3, mean ± s.d.). **h**–**j**, Conjugating activity of recombinant GST-U19 (**h**), U24 (**i**) and U25 (**j**) on DNAN and TNT substrates to GSH. TNT-conjugate peaks are labelled on the traces as previously identified^8,9^. **j**, Inset: expanding the A_220_ *y* axis revealed ~100-fold smaller DNAN-conjugate peak for U25. No peaks were present in DMSO controls. HPLC analysis was repeated on 3 separate reactions. *P < 0.05, **P < 0.01, ***P < 0.001, ****P < 0.0001. All data and *P* values can be found in the Source data file.

**Extended Data Fig. 2:**
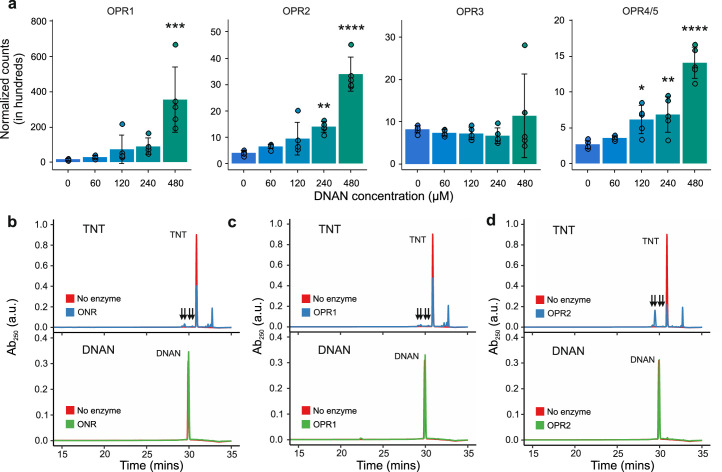
Relative expression of oxophytodienoate reductases (OPRs) in response to DNAN, and activity towards TNT and DNAN. (**a**) Upregulation of OPRs from a transcriptomics study of two-week-old, liquid culture grown plants dosed with DNAN for 6 hours (n = 5, mean ± SD). Statistical analysis was performed by one-way ANOVA and Tukey’s post hoc test. * p < 0.05, ** p < 0.01, *** p < 0.001, **** p < 0.0001. HPLC analysis of the activity of purified recombinant (**b**) pentaerythritol tetranitrate reductase from *Enterobacter cloacae* (ONR), (**c**) OPR1 and (**d**) OPR2 towards DNAN and TNT. HPLC analysis was repeated on three separate reactions. All the data and p values can be found in the Source data file.

The detection of DNAN transformation intermediates here and in ref. ^10^ indicates that plants could detoxify DNAN through enzymatic pathways. To understand the genetic basis behind this, a transcriptomics analysis was performed. Arabidopsis seeds were germinated, then grown in liquid culture for 14 days, dosed for 6 h with 0, 60, 120, 240 or 480 µM DNAN, then the RNA extracted. Unsurprisingly, the highest concentration of DNAN induced the most significant differential expression, with 3,629 transcripts significantly (*P* < 0.001) upregulated (Extended Data Table 1). In agreement with published studies for TNT^8,11,12^, genes that were the most upregulated included those involved in nitroreduction (oxophytodienoate reductases, *OPR*s)^9^, conjugation (uridine 5′-diphospho-glucuronosyltransferases, *UGT*s, and glutathione transferases, *GST*s)^8,13,14^ and transport (ATP binding cassette transporters; multidrug resistance-associated protein 2, *MRP2*). TNT detoxification steps in plants mainly occur at the nitro groups, with initial transformation catalysed by OPRs to form hydroxyl-amino-dinitrotoluenes (HADNT) and then amino-dinitrotoluenes (ADNT)^9^. In our transcriptomics study, *OPR1* and *2* were significantly upregulated in response to DNAN (Fig. 2a and Extended Data Fig. 2a), suggesting that the encoded enzymes could also be involved in its transformation. To test for transformation activity, recombinant, purified OPR1 and OPR2 were assayed. Pentaerythritol tetranitrate reductase (ONR) from *Enterobacter cloacae* was also included as this member of the Old Yellow Enzyme family and homologue of the OPRs has been shown to nitroreduce TNT^9,15^. As in our previous studies, we confirmed activity of ONR and OPR1 and 2 on TNT; however, much lower levels of activity were observed for DNAN (Fig. 2c and Extended Data Fig. 2b), indicating that OPRs have only low activity towards DNAN in planta.

**Extended Data Fig. 3:**
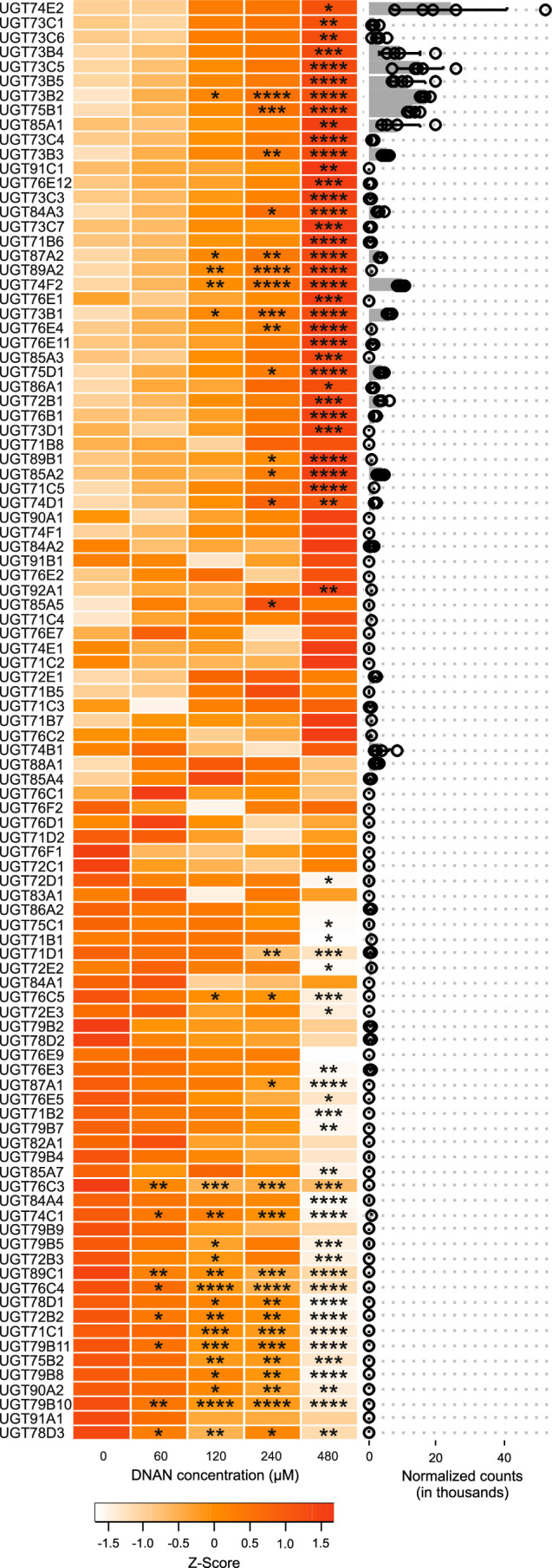
Expression of uridine 5′-diphospho-glucuronosyltransferases (UGTs) in response to DNAN. Upregulation of UGTs from a transcriptomics study of two-week-old, liquid culture grown plants dosed with DNAN for 6 hours (n = 5, mean ± SD). Heatmap showing the relative expression levels across different concentrations of DNAN, in terms of z-score Source data.

The HADNT and ADNT transformation intermediates of TNT can be conjugated at either the 2- or 4- isomer positions to sugars by UGTs^8^. In our transcriptomics data, a substantial number of *UGT* genes were upregulated (Extended Data Fig. 3). We did not detect glucose conjugates of 2-ANAN using high-performance liquid chromatography (HPLC), which is probably because the activity of OPRs catalysing the conversion of DNAN to transformation intermediates is much lower than that seen for TNT.

Direct conjugation of TNT can also occur, either through the methyl group, or by substitution of a nitro group, for sulfur by the Tau-class GSTs^13,14^. Tau-class GSTs are involved in the detoxification of many other xenobiotics including herbicides^16^. The addition of a hydrophilic conjugate, such as a glutathione or sugar moiety, to a xenobiotic compound decreases the hydrophobicity of the parent compound. This conjugated form is unable to passively cross biological membranes, allowing for controlled localization using active transport. Evidence suggests that TNT conjugates are stored in the vacuole and apoplast before their incorporation into cellular macromolecular structures such as lignin^1^. Given our understanding of the detoxification of TNT, it might be expected that DNAN would also be subject to similar detoxification routes in planta. Indeed, glutathione-DNAN and glycosylated conjugates, albeit at low amounts, have been described in Arabidopsis for DNAN^10^.

**Extended Data Fig. 4:**
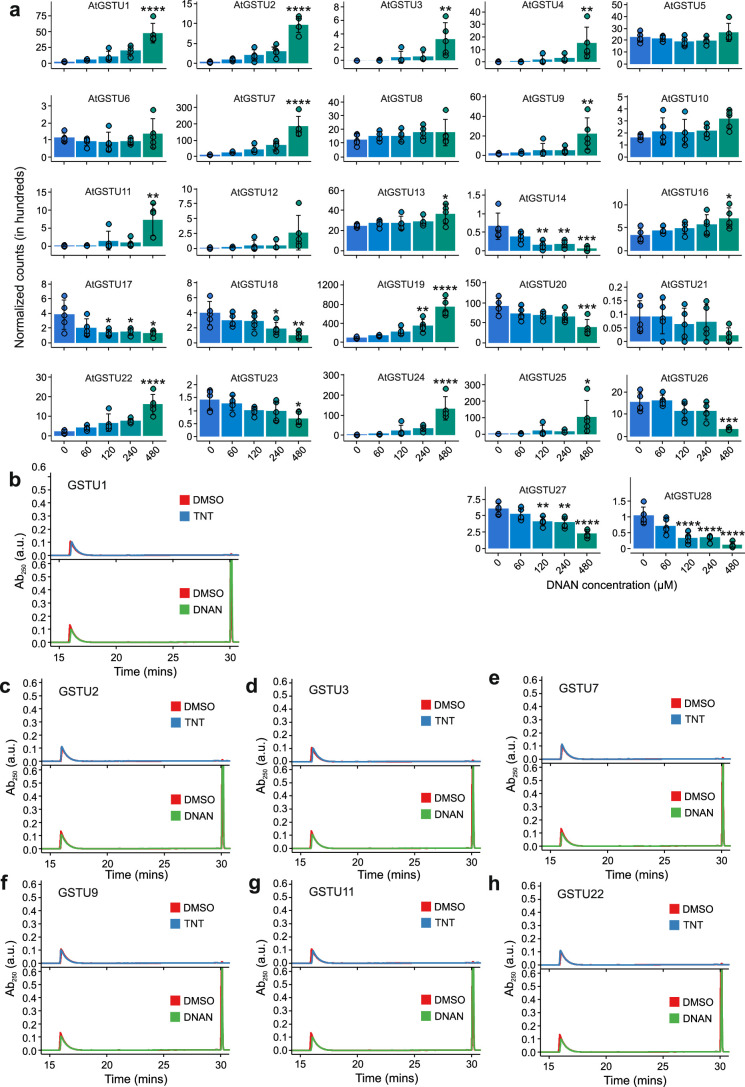
Relative expression and activity of Tau class glutathione reductases (GSTs) in the presence of DNAN. (**a**) Upregulation of GSTs from a transcriptomics study of two-week-old, liquid culture grown plants dosed with DNAN for 6 hours (n = 5, mean ± SD). Statistical analysis was performed by one-way ANOVA and Tukey’s post hoc test. * p < 0.05, ** p < 0.01, *** p < 0.001, **** p < 0.0001. HPLC analysis of the conjugating activity of recombinant GST: (**b**) U1, (**c**) U2, (**d**) U3, (**e**) U7, (**f**) U9, (**g**) U11, and (**h**) U22 to GSH. Both DNAN and TNT were tested. TNT-conjugate peaks are labelled on the trace as previously identified^7,8^. No peaks were present in DMSO controls or DNAN substrate reactions. HPLC analysis was repeated on three separate reactions. All the data and p values can be found in the Source data file.

Our transcriptomic analysis indicated that a suite of Tau-class *GST* genes is upregulated in response to DNAN treatment (Fig. 2b and Extended Data Fig. 4a). To investigate the identities of conjugation products, tissue extracts from 2-week-old, hydroponically grown Arabidopsis plants dosed with 250 µM DNAN or TNT were analysed using HPLC. In agreement with previous studies^8,9,13,14^, conjugation peaks were absent from shoot but clearly identified for TNT-dosed root extracts. However, conjugation peaks were not seen in the DNAN-dosed extracts from plant roots or shoots (Fig. 2d,e). A lack of identifiable conjugation peaks could be due to the sensitivity of our assay techniques, as previous studies used stable isotope labelled DNAN^10^. Thus, to test conjugation activity directly, we cloned, expressed and purified the nine most upregulated GSTs in our transcriptomic study (Fig. 2f).

To test for activity, each recombinant GST was incubated with glutathione (GSH) and 1-chloro-2,4-dinitrobenzene (CDNB), a model substrate for GSTs. The GSTs U25, U24 and U19, the most upregulated GSTs in response to TNT^13,14^, exhibited the highest activities towards CDNB (Fig. 2g) and were thus tested for activity towards either DNAN or TNT (Fig. 2h–j). The HPLC traces of GST-U24 and U25 contained relatively small but identifiable peaks that corresponded to conjugated TNT transformation products. Expanding the A_220_ *y* axis revealed a ~100-fold smaller DNAN-conjugate peak for U25 (Fig. 2j), suggesting that these GSTs have a much lower affinity for DNAN and may not play a notable physiological role in DNAN detoxification. We also demonstrated the conjugation of TNT catalysed by GST-U19. Conjugation peaks were not detected for either compound when incubated with GST-U1, U2, U3, U7, U9 or U22 (Extended Data Fig. 4b–h).

**Extended Data Fig. 5:**
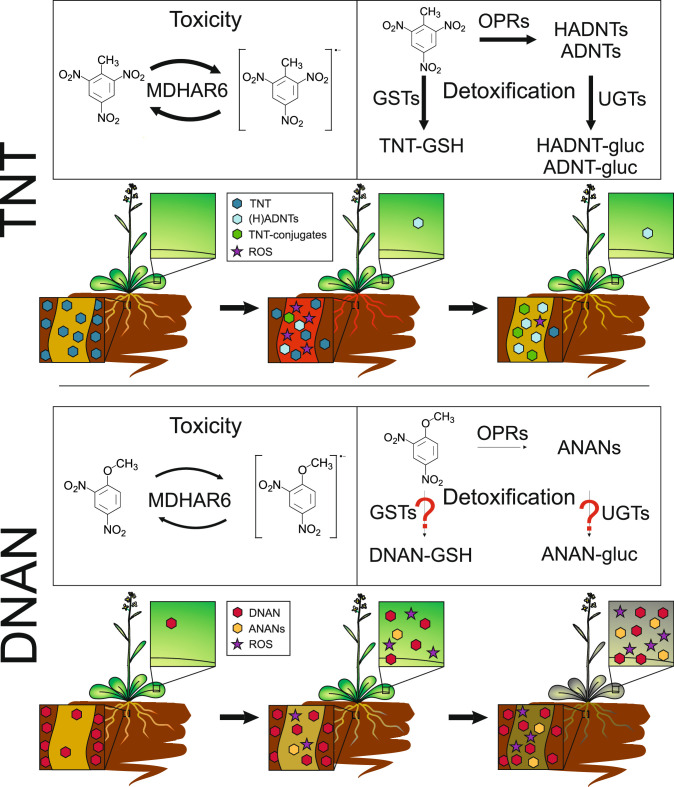
Schematic illustrating the rapid uptake and metabolism of TNT in root tissue (acute toxicity) verses slower uptake and slow metabolism of DNAN in root and aerial tissue (chronic toxicity). Appearance of plant post-exposure is dose-dependent and not shown here. Reaction arrows are wider for TNT to indicate the higher activity of transforming and conjugating enzymes (OPRs, GSTs and UGTs) towards TNT over DNAN. Question marks indicate possible activity by these enzymes. ROS – reactive oxygen species.

As the Arabidopsis genome encodes 48 *GST* and 107 *UGT* genes, it is likely that the low levels of DNAN conjugates observed here and reported previously^10^ could also be produced at low levels by endogenously expressed genes. Together, the results presented here show that while TNT causes acute root-localized toxicity, this is quickly mitigated by immobilization within the root via a series of transformation and conjugation steps (Extended Data Fig. 5). In contrast, the reduced ability to transform and conjugate DNAN results in extended exposure to MDHAR6, the key determiner of toxicity in Arabidopsis. Moreover, this relatively low conjugation ability means that the DNAN remains mobile, accumulating in both root and shoot tissue, exposing DNAN to herbivores and the wider food chain, and risking further environmental implications. Effective remediation strategies are needed to ensure that this xenobiotic does not repeat the environmental legacy left by its predecessor, TNT.

## Methods

### Materials

The TNT was obtained from Dstl Fort Halstead, 2-ADNT and 4-ADNT were sourced from Cole-Parmer. DNAN was purchased from Avocado Research Chemicals, and 2-ANAN, 4-ANAN and DAAN were sourced from Thermo Fisher. Following our previous studies, MDHAR6 was expressed from a pET52b vector, and GST-U24 and GSTU25 expressed from pET-YSBLIC3C vectors^3,14^. Genes encoding GST-U1, 2, 4, 7, 9, 19 and 22 were obtained in pET100 expression vectors (GeneArt). Genes encoding OPR1 and 2 were in pET16b vectors, following our previous study^9^. All plant experiments were carried out using Arabidopsis ecotype Columbia-0. *mdhar6-1* knockout lines were obtained as outlined in our previous study^3^.

### DNAN phytotoxicity studies

Sterile, stratified Arabidopsis seeds were germinated for 24 h on 1/2 MS agar^17^. Eight seedlings were transferred to shake flasks with 20 ml 1/2 MS media plus 1% sucrose. Flasks were incubated on a rotary shaker at 100 revolutions per minute (r.p.m.) at 21 °C in 20 μmol m^−2^ s^−1^ light with a 16-h photoperiod. After 2 weeks, media were replenished and dosed with either 0, 50, 125 or 250 μM of DNAN or TNT dissolved in dimethylsulfoxide (DMSO). Controls that had not been dosed with xenobiotic were spiked with the equivalent concentration of DMSO. At 7 days post dosing, tissue was collected, washed and weighed.

### HPLC analysis

Reactions were analysed by HPLC using a Waters HPLC system (Waters 2695 separator and Waters photodiode array detector) with a Waters X-Bridge C18 column (300 × 4.5 mm, 5 μm). The mobile phases for the gradient conditions were as follows: mobile phase A, acetonitrile; mobile phase B, water plus 0.1% (v/v) formic acid. The gradient ran as follows: 0 min, 5% A and 95% B; 5 min, 5% A and 95% B; 25 min, 40% A and 60% B; 30 min, 100% A and 0% B; and 35 min, 5% A and 95% (v/v) B. Data were collected and analysed in Empower 3 Pro software and visualized in R^18^.

### Recombinant protein production

MDHAR6 was expressed in *E. coli* Arctic Express as previously reported^3^. GST-U24 and GSTU25 were expressed in *E. coli* BL21(DE3) as previously reported^14^. The remaining GSTs were expressed in *E. coli* BL21 pLysS(DE3). OPRs/ONR were expressed in *E. coli* Rosetta-Gami 2(DE3). Briefly, transformed colonies were selected from LB agar plates containing the relevant antibiotic selection marker and grown in 50 ml LB cultures overnight. For expression of all GSTs and OPRs/ONR, 10 ml of these cultures was then inoculated into 500 ml autoinduction media with antibiotic. Cultures were incubated at 37 °C throughout the day and transferred to 20 °C overnight, shaking at 180 r.p.m. throughout. For expression of MDHAR6, 10 ml of culture was inoculated into 500 ml LB with antibiotic and incubated at 37 °C, shaking at 250 r.p.m., until the cell density reached an optical density at 600 nm (OD_600_) of ~0.6. Next, 0.6 mM isopropyl β-d-1-thiogalactopyranoside was added to induce expression of MDHAR6 and the culture incubated at 15 °C, shaking at 180 r.p.m. for 24 h. Cultures were pelleted at 4,000 *g* for 15 min at 4 °C and pellets stored at −20 °C.

Recombinant MDHAR6 was purified using a StrepTrap column (GE Healthcare) as previously reported^3^. Bacterial pellets were resuspended in 50 mM NaH_2_PO_4_ (pH 8), 300 mM NaCl, 0.1% Tween-20 and 70 μl 0.1 M phenylmethanesulfonylfluoride in isopropanol. Recombinant MDHAR6 was eluted off the column using the same buffer with 2.5 mM desthiobiotin. Recombinant GST-U24 and GSTU25 were also purified using a Glutathione Sepharose 4B resin (GE Healthcare) as previously reported^14^. The remaining recombinant GSTs and OPRs/ONR were purified using a HisTrap column (GE Healthcare). GST bacterial cell pellets were resuspended in PBS (pH 7.4), whereas OPR/ONR bacterial cell pellets were resuspended in 50 mM potassium phosphate (pH 7.5). Recombinant GSTs and OPRs/ONR proteins were eluted off the column using a gradient of up to 10 mM reduced glutathione and 500 mM imidazole, respectively.

### MDHAR6 activity assays

The activity of MDHAR6 towards DNAN and TNT was confirmed through the oxidation of NADH, monitored at 340 nm. Reactions contained 74 μg ml^−1^ MDHAR and 100 μM NADH in 50 mM Tris, 1 mM EDTA (pH 7.6) and 10% DMSO. Reactions were initiated with the addition of 500 μM TNT or DNAN. Data were collected on the Cary 50 WinUV Sime Reads software, analysed and visualized in R^18^.

### Soil studies

The soil studies were conducted as previously described^3^. Briefly, 5-day-old seedlings were planted into pots containing soil contaminated with a specified concentration of DNAN. Seedlings were then grown with 180 μmol m^−2^ s^−1^ light with a 12-h photoperiod. Temperatures were set to 21 °C during light and 18 °C during dark conditions. After 5 weeks of growth, aerial tissue was harvested and weighed.

### Methanol extractions

Fresh plant tissues were ground in liquid nitrogen and metabolites were extracted using 1 ml methanol per 10 mg fresh weight. After centrifugation, the supernatant was collected, evaporated and resuspended in methanol:water (20:80) at a concentration of 1 µl mg^−1^ fresh weight. Extracts were analysed using HPLC.

### Hydroponic experiments

Hydroponic experiments were established to determine the location and toxicity of DNAN and its transformation products within Arabidopsis plants. Sterile seedlings were stratified and grown on 1/2 MS agar for 10 days. These were then transferred to sponges cut into 2 × 2 cm along the radius and inserted into corresponding cut-outs in polystyrene rafts. Rafts, each containing six sponges, were floated in tip boxes containing 200 ml 1/2 MS. The hydroponic plants were incubated under the same light and temperature conditions as the shake flasks, with media replenished weekly. After 4 weeks of incubation, boxes were dosed with 250 μM TNT or DNAN dissolved in DMSO. Negative controls were spiked with the equivalent concentrations of DMSO. Plants were harvested at 4 days post dosing. Roots and shoots were separated, washed and weighed before methanol extractions, and analysed by HPLC.

### Agar plate experiments

Sterile, stratified Arabidopsis seeds were imbibed for 2–3 days at 4 °C and then spotted onto 1/2 MS agar in square plates. The 1/2 MS agar was dosed with varying concentrations of DNAN, 2-ANAN, 4-ANAN, DAAN or DMSO as a control. Plates were placed vertically in a growth room under the same light and temperature conditions as for the shake flasks. Seedling root lengths were measured using ImageJ^19^ and the biomass weighed.

### Transcriptomics analysis

Arabidopsis seedlings were incubated in shake flasks containing ½ MS growth media for 14 days and then dosed for 6 h with 0, 60, 120, 240 or 480 µM DNAN. Five biological replicates were performed for each treatment. The RNA was collected from plant tissue that had been ground in liquid nitrogen using EasyPure plant RNA kits (Transgen Biotech) and assessed using TapeStation (Aligent) gel-capillary electrophoresis. Levels of RNA degradation were found to be minimal and the quantity of RNA sufficient for sequencing. Consequently, polyA tail selection was performed to enrich for messenger RNA (mRNA) using the NEBNext Poly(A) mRNA Magnetic Isolation Module and NEBNext Ultra II Directional RNA Library Prep kit (New England Biolabs), and these samples sequenced using an Illumina HiSeq 3000 platform. The resultant 370 million paired-end fragments were mapped to the TAIR 10 Arabidopsis cDNA reference library^20^ using BWA software and raw counts outputted using HTSeq. Normalization and data visualization were carried out in R using the DESeq package^18,21^. Filtering was applied to the expression matrix to include only sequences present at over ten counts in at least three biological replicates. Genes were annotated using the classification provided by The Gene Ontology Consortium (2018), and gene families were obtained from TAIR (https://www.arabidopsis.org/browse/genefamily/index.jsp).

### OPR activity assays

Activity was determined by measuring oxidation of NADH on a spectrophotometer at 340 nm. Reaction conditions were as follows: 50 mM potassium phosphate buffer (pH 7.5), 120 μM TNT/DNAN and 200 μM NADH. Reactions were initiated by addition of 50 μl enzyme to the cuvette, for a final concentration of 10 μg ml^−1^, made up to a total of 1 ml. Data were collected on the Cary 50 WinUV Sime Reads software, analysed and visualized in R^18^.

### GST-conjugate analysis

Seedlings were germinated and transferred to the hydroponic system, as already outlined, dosed with 250 μM TNT or DNAN. To determine whether TNT- or DNAN-GSH conjugates were present, plant tissue was methanol extracted and analysed by HPLC, as outlined above.

### Glutathione transferase assays

Purified recombinant GSTs were assayed against CDNB to confirm that the enzymes were active. Reactions were carried out as previously reported^13^. Briefly, reactions contained 100 mM potassium phosphate (pH 6.5), 25 μg GST, 5 mM GSH and 1 mM CDNB. Reactions were made up to 1 ml, incubated at 20 °C and measured spectrophotometrically at 340 nm. Data were collected on the Cary 50 WinUV Sime Reads software, analysed and visualized in R^18^.

Purified recombinant GST-U1, 2, 4, 7, 9, 19, 22, 24 and 25 enzymes were assayed for conjugation activity against DNAN. Reaction conditions were as follows: 100 mM potassium phosphate (pH 7.0), 5 mM GSH and 0.2 mM TNT/DNAN. Each reaction also contained 400 μg ml^−1^ of each GST enzyme. Reactions were stopped by mixing 80 μl of reaction mixture with 20 μl 50% trichloroacetic acid. Precipitates were removed by centrifugation and the remaining supernatant analysed by HPLC.

### Statistical analysis

One-way analysis of variance (ANOVA) and two-sided Student’s *t*-test where indicated were performed using R base packages^18^. Data analysis by ANOVA was followed by Tukey’s post hoc test. *P < 0.05, **P < 0.01, ***P < 0.001, ****P < 0.0001.

### Reporting summary

Further information on research design is available in the Nature Portfolio Reporting Summary linked to this article.

## Supplementary information

Reporting Summary

## Source data

Source Data Statistical source data and Fig. 1b unprocessed gel lanes.

## Figures and Tables

**Extended Data Table 1:**
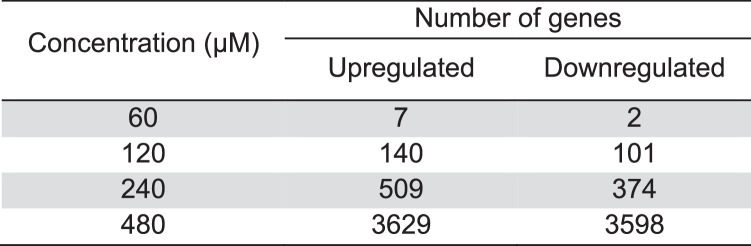
The number of genes differentially expressed in Arabidopsis plants exposed to different concentrations of DNAN

## Data Availability

The RNA transcriptomic data generated in this manuscript have been deposited in the NCBI’s Gene Expression Omnibus (GEO) under the accession code ‘GSE264500’. The transcriptomic reads were mapped to the TAIR 10 Arabidopsis cDNA reference library. Genes were annotated using classifications from the Gene Ontology Consortium. Gene families were obtained from TAIR. Data presented in this manuscript can be found in the source data file. Source data are provided with this paper.
