# Supplementary material for: New weapons explosive exhibits persistent toxicity in plants
Source: Nat Plants. 2024 Nov 28;11(1):16–22. doi: 10.1038/s41477-024-01863-0 (PMC11757145; doi:10.1038/s41477-024-01863-0)
Supplement: Supplementary file 1 — Reporting Summary [file 41477_2024_1863_MOESM1_ESM.pdf]

nature portfolio

Corresponding author(s): Elizabeth Rylott, Neil Bruce

Last updated by author(s): Oct 17, 2024

Reporting Summary

Nature Portfolio wishes to improve the reproducibility of the work that we publish. This form provides structure for consistency and transparency in reporting. For further information on Nature Portfolio policies, see our Editorial Policies and the Editorial Policy Checklist.

Statistics

For all statistical analyses, confirm that the following items are present in the figure legend, table legend, main text, or Methods section.

n/a	Confirmed	
☐	☒	The exact sample size (*n*) for each experimental group/condition, given as a discrete number and unit of measurement
☐	☒	A statement on whether measurements were taken from distinct samples or whether the same sample was measured repeatedly
☐	☒	The statistical test(s) used AND whether they are one- or two-sided<br>*Only common tests should be described solely by name; describe more complex techniques in the Methods section.*
☐	☒	A description of all covariates tested
☐	☒	A description of any assumptions or corrections, such as tests of normality and adjustment for multiple comparisons
☐	☒	A full description of the statistical parameters including central tendency (e.g. means) or other basic estimates (e.g. regression coefficient) AND variation (e.g. standard deviation) or associated estimates of uncertainty (e.g. confidence intervals)
☐	☒	For null hypothesis testing, the test statistic (e.g. F, t, r) with confidence intervals, effect sizes, degrees of freedom and P value noted<br>*Give P values as exact values whenever suitable.*
☒	☐	For Bayesian analysis, information on the choice of priors and Markov chain Monte Carlo settings
☒	☐	For hierarchical and complex designs, identification of the appropriate level for tests and full reporting of outcomes
☒	☐	Estimates of effect sizes (e.g. Cohen's d, Pearson's r), indicating how they were calculated

Our web collection on statistics for biologists contains articles on many of the points above.

Software and code

Policy information about availability of computer code

Data collection	HPLC: Empower 3 Pro software version (Waters); Spectrophotometry: Cary 50 WinUV Simple Reads Application software version 3.0 (Agilent)
Data analysis	HPLC: Empower 3 Pro software (Waters); Root lengths: ImageJ version 1.54d (NIH); Transcriptomics: BWA software version 0.7.17, HTSeq version 0.13.5 software, DESeq2 package version 3.19 (R Bioconductor); statistics and data plotting: R (version 4.4.1) packages in RStudio (version 2024.09.0)

For manuscripts utilizing custom algorithms or software that are central to the research but not yet described in published literature, software must be made available to editors and reviewers. We strongly encourage code deposition in a community repository (e.g. GitHub). See the Nature Portfolio guidelines for submitting code & software for further information.

Data

Policy information about availability of data

All manuscripts must include a data availability statement. This statement should provide the following information, where applicable:

- Accession codes, unique identifiers, or web links for publicly available datasets
- A description of any restrictions on data availability
- For clinical datasets or third party data, please ensure that the statement adheres to our policy

The RNA transcriptomic data generated in this manuscript have been deposited in the NCBI's Gene Expression Omnibus (GEO) under the accession code "GSE264500" (https://www.ncbi.nlm.nih.gov/geo/query/acc.cgi?acc=GSE264500). The TAIR 10 Arabidopsis cDNA reference library was used in the transcriptomic

analyses (https://www.ncbi.nlm.nih.gov/datasets/genome/GCF_000001735.3/). Genes were annotated using the classification of The Gene Ontology Consortium (https://geneontology.org/). Gene families were obtained from TAIR (https://www.arabidopsis.org/browse/genefamily/index.jsp). Data presented in this manuscript can be found in the source data file.

Research involving human participants, their data, or biological material

Policy information about studies with human participants or human data. See also policy information about sex, gender (identity/presentation), and sexual orientation and race, ethnicity and racism.

Reporting on sex and gender	N/A
Reporting on race, ethnicity, or other socially relevant groupings	N/A
Population characteristics	N/A
Recruitment	N/A
Ethics oversight	N/A

Note that full information on the approval of the study protocol must also be provided in the manuscript.

Field-specific reporting

Please select the one below that is the best fit for your research. If you are not sure, read the appropriate sections before making your selection.

☒ Life sciences ☐ Behavioural & social sciences ☐ Ecological, evolutionary & environmental sciences

For a reference copy of the document with all sections, see nature.com/documents/nr-reporting-summary-flat.pdf

Life sciences study design

All studies must disclose on these points even when the disclosure is negative.

Sample size	Sample sizes were chosen so that significant and meaningful comparisons could be carried out between conditions in statistical analyses. Enzymatic assays were carried out in at least triplicate and experiments involving plants had at least five replicates to allow for this.
Data exclusions	Data was excluded if there was technical error on data acquisition. In the case of the plant work, data was also excluded if microbial contamination resulted in stunted growth or death of the plants.
Replication	Biological replicates for plant experiments were collected on independently germinated seeds and independent enzyme reactions. The only exception is Supplementary Fig. 1a where the 250 µM DNAN day 6 time point is n = 1 due to an error in acquiring the final time course samples. HPLC chromatograms presented represent a single sample of a number of replicates stated in the Figure legend.
Randomization	Material samples and healthy plants were allocated randomly into experimental groups.
Blinding	No blinding was used in this study as this was not relevant to the biochemical enzyme reactions. Germinated seeds were randomly allocated for each of the experimental conditions.

Reporting for specific materials, systems and methods

We require information from authors about some types of materials, experimental systems and methods used in many studies. Here, indicate whether each material, system or method listed is relevant to your study. If you are not sure if a list item applies to your research, read the appropriate section before selecting a response.

Materials & experimental systems

n/a	Involved in the study	
☒	☐	Antibodies
☒	☐	Eukaryotic cell lines
☒	☐	Palaeontology and archaeology
☒	☐	Animals and other organisms
☒	☐	Clinical data
☒	☐	Dual use research of concern
☐	☒	Plants

Methods

n/a	Involved in the study	
☒	☐	ChIP-seq
☒	☐	Flow cytometry
☒	☐	MRI-based neuroimaging

Plants

Seed stocks	Arabidopsis thaliana Columbia-0 ecotype seeds were obtained from the Nottingham Arabidopsis Stock Centre (NASC).
Novel plant genotypes	mdhar6-1 mutant plant lines were generated as we have previously reported (Johnston et al, 2015).
Authentication	mdhar6-1 mutant plant lines were generated and mutation mapped as previously reported (Johnston et al, 2015).
